# Tradition and Innovation in Raw Meat Products with a Focus on the Steak Tartare Case

**DOI:** 10.3390/foods14132326

**Published:** 2025-06-30

**Authors:** Giovanni D’Ambrosio, Francesca Maggio, Annalisa Serio, Antonello Paparella

**Affiliations:** Department of Bioscience and Technology for Food, Agriculture and Environment, University of Teramo, Via Balzarini 1, 64100 Teramo, Italy; giovanni.dambrosio@studenti.unite.it (G.D.); fmaggio@unite.it (F.M.); aserio@unite.it (A.S.)

**Keywords:** steak tartare, microbiology, shelf life, technology, nitrite, antimicrobials

## Abstract

Steak tartare is a ready-to-eat (RTE) meat product, prepared with finely chopped or ground raw beef, with a rich culinary history and increasing consumption trend in the last years. Yet, its microbiological safety and technological challenges remain largely under-investigated. This review analyses the regulations, the safety, and technological advances in steak tartare manufacturing, focusing on microbiological risks due to potential contamination by pathogens like *Salmonella* spp., *Listeria monocytogenes*, and *Escherichia coli* O157:H7. From this perspective, the outbreaks associated with the consumption of raw meat products have confirmed the importance of good hygiene practice and process control, currently based on the presence of nitrite in the formulation and accurate cold chain management. Recently, the EU regulations have set stricter limits for the use of nitrites and nitrates in meat products, and this evolution has increased the interest in natural alternatives. The scientific literature indicates that plant-based antimicrobials, high-pressure processing (HPP), and novel starter cultures can be promising tools to improve raw meat safety and shelf life. This review analyses the possible options for nitrite replacement, which might involve combined interventions with natural antimicrobials, starter cultures, and packaging solutions. Future studies need to address the microbial behaviour and dynamics in nitrite-free formulations, including safety validation by challenge testing with foodborne pathogens. In this respect, steak tartare could be a model for innovation in the meat industry. However, considering the challenges that must be faced, collaboration across disciplines will be essential to meet regulatory constraints and consumer expectations while ensuring product quality and safety.

## 1. Introduction

The first diffused information about steak tartare was reported in the 17th century by Guillaume Levasseur de Beauplan, a French engineer and cartographer [1]. The original “recipe”, attributed to the Crimean Tatars, an East European Turkic ethnic group, consisted of sliced salted horse meat, put under the steed’s saddle for a couple of hours, and then chopped. However, the first written recipe dates to the 19th century, with Farmer Fannie Merritt’s “Boston Cooking-School Cookbook” (1896) [2], where steak tartare is called “Hamburg steak”. The recipe clearly states the addition of several ingredients: salt, pepper, onion juice or chopped shallot, nutmeg, and eggs.

In 1903’s *Le Guide Culinaire* from Georges Auguste Escoffier, steak tartare is mentioned both as “Bifteck à l’Américaine” and “Bifteck à la Tartare” [3]. The Bifteck à l’Américaine is prepared with a lean beef slice, with the nerves and fat removed, finely chopped and formed on a plate with a small cavity in the centre where an egg yolk is placed, accompanied by separate capers, chopped onions, and parsley. Instead, in the Bifteck à la Tartare, the steak is prepared “as for the Américaine”, but without egg yolk on top, and accompanied by tartare sauce on the side.

The same distinction and way of preparation is found in the *Larousse Gastronomique Cookbook* (1938) [4], and a similar codification is used in Italian cuisine, which differentiates “Tartare di manzo”, usually served with different ingredients (capers, cornichons, raw egg yolk, etc.), from “Battuta di manzo”, served only with olive oil, salt, and pepper. Also, *Luchow’s German Cookbook* (1952) [5] distinguishes “Luchows’s beef steak tartar” from “Raw meat Lucullus Schlemmerschnitte”, which was a version of beef steak tartare served on toast garnished with capers, raw eggs, onions, caviar, etc.

Today, steak tartare is valued for its texture, flavour, richness in nutrients, and cultural significance in many cuisines. However, its raw nature poses significant microbiological risks, which are usually managed by focusing the attention on the cold chain and the use of additives in the formulation. Among these additives, nitrite, ascorbic acid, and citric acid are the most used to stabilise the colour and control the growth of foodborne pathogens. Although this product is widespread in the meat market, only a few studies have been carried out on its technological, chemical, and microbiological characteristics. Therefore, understanding the microbiological hazards and the technological strategies for the safe production of steak tartare is of paramount importance. Steak tartare is one of the most successful products of its category. In fact, although beef production has declined in several countries and particularly in the European Union [6], the global raw meat market is expected to grow from USD 1252.61 billion in 2024 to USD 1763.69 billion in 2034, at a compound annual growth rate (CAGR) of 6.8%, which is attributed also to the specific growth of novel sustainable products in the health and wellness sector [7]. Hence, steak tartare can be considered a case study to investigate current manufacturing strategies and future innovation in the manufacturing of raw meat products.

Although steak tartare is a well-established and commercially relevant raw meat product, particularly in European gastronomy, comprehensive scientific reviews on its microbiological risks, processing technologies, additives, and innovative preservation strategies are still lacking. Therefore, this review aims to critically analyse the scientific evidence on the technology and microbiology of steak tartare, including its microbial ecology, major outbreaks linked to its consumption, current additive use, and emerging trends in formulation. To identify the relevant literature, key issues were first defined—such as raw materials, recipes, processing conditions, and additive substitution. Scientific publications were then retrieved through various search engines (Google Scholar, PubMed, and ResearchGate) up to 15 May 2025, using keywords including “steak tartare,” “steak haché,” “beef tartare,” “microbiology,” “technology,” “processing,” “safety,” “shelf life,” “nitrite,” “antimicrobials,” and “innovation.” The selected literature was screened and categorised by relevance, highlighting commonalities and divergences among sources. This review addresses the existing knowledge gap by providing a comprehensive overview and identifying future directions, positioning steak tartare as a model for innovation in the safety and formulation of raw meat products.

## 2. Regulatory Framework

Differently from the steak tartare that is prepared at home or in the restaurants, which does not contain additives and has a short shelf life, industrially produced steak tartare is normally sold in a Vacuum Skin Packaging (VSP) with a mean shelf life of around 12 days, ensured by a set of unit operations including the use of additives. Therefore, according to Regulation (EC) No. 853/2004 [8], the industrial steak tartare is defined as a “meat product”, meaning that its final appearance results from the “processing of meat or from the further processing of such processed products, so that the cut surface shows that the product no longer has the characteristics of fresh meat”. In detail, the technological treatments that cause a substantial change in the characteristics of fresh meat are defined in art. 2 of Regulation (EC) No. 852/2004 [9] and include “heating, smoking, curing, maturing, drying, marinating, extraction, extrusion or a combination of those processes”. The classification as “meat product” and not “meat preparation” is confirmed by the evidence that nitrite, the most common additive in the industrial steak tartare, is not permitted by Regulation (EC) No. 1333/2008 [10] in meat preparations.

**Figure 1:**
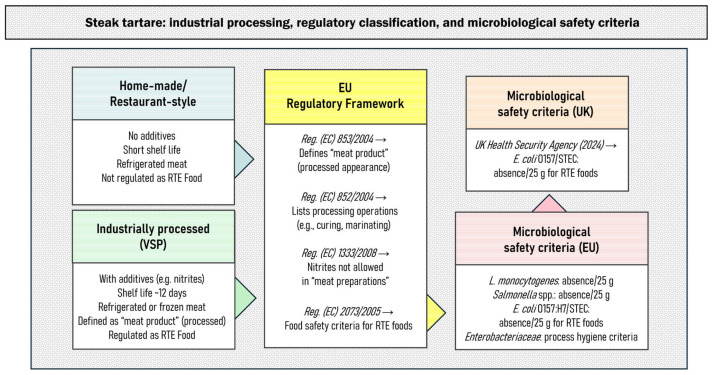
Regulatory framework and microbiological safety criteria for industrial steak tartare in the EU and UK.

The regulatory framework for steak tartare (Figure 1) also includes Regulation (EC) No. 2073/2005 [11], according to which steak tartare is classified as a “ready-to-eat food able to support the growth of *Listeria monocytogenes*, other than those intended for infants and for special medical purposes” and “minced meat and meat preparations intended to be eaten raw”. The compulsory food safety criterion for *L. monocytogenes* is the absence in 25 g, as its pH and a_w_ values support the growth above the set criterion of 2 Log CFU/g. The same section on food safety criteria requires the absence of *Salmonella* spp. in 25 g of meat products intended to be eaten raw, whereas *E. coli* O157:H7 or other Shigatoxin-producing (STEC)/Enterohemorrhagic *E. coli* (EHEC) serotypes are set as food safety criterion only for ready-to-eat (RTE) sprouts. As for the process hygiene criteria according to EU regulation, only *Enterobacteriaceae* and *Escherichia coli* are considered for fresh meat and meat products.

Among the possible future food safety criteria in the EU, *E. coli* O157:H7 and STEC serotypes are extremely important, as these pathogens are commonly isolated from the gastrointestinal tract of ruminants (including cattle, sheep, goats, and other farmed animals). Therefore, a zero-tolerance criterion was adopted for *E. coli* O157:H7 in raw beef products in the USA.

In this respect, in the EU, based on an opinion of the Scientific Committee on Veterinary Measures relating to Public Health on Verotoxigenic *E. coli* (VTEC) in foodstuffs [12], microbiological guidelines and corrective actions aimed at reducing faecal contamination along the food chain (e.g., testing for *Enterobacteriaceae*) already contribute to a reduction of a VTEC-associated public health risk. According to this opinion, applying end-product microbiological standards for VTEC O157 would not deliver meaningful reductions in the risk for the consumer, due to the sporadic occurrence and low prevalence of VTEC O157 found in food commodities. This does not mean that this microorganism is considered unimportant, but only that process control is best achieved by setting a criterion for an indicator group of microorganisms such as *Enterobacteriaceae* or generic *E. coli*.

Finally, the United Kingdom (UK) Health Security Agency (2024) [13], after the UK’s separation from the EU, adopted a food safety criterion for the absence of *E. coli* O157 and other STEC in 25 g of all RTE foods placed on the market.

## 3. Industrial Processing

The production process of steak tartare closely resembles that of ground beef patties, although flow charts, machinery, and process parameters (e.g., dimension of the meat particles, time and rpm of mixing, types of machinery used to mix the ingredients in a vacuum environment, etc.) can change according to the recipe and the manufacturer.

**Figure 2:**
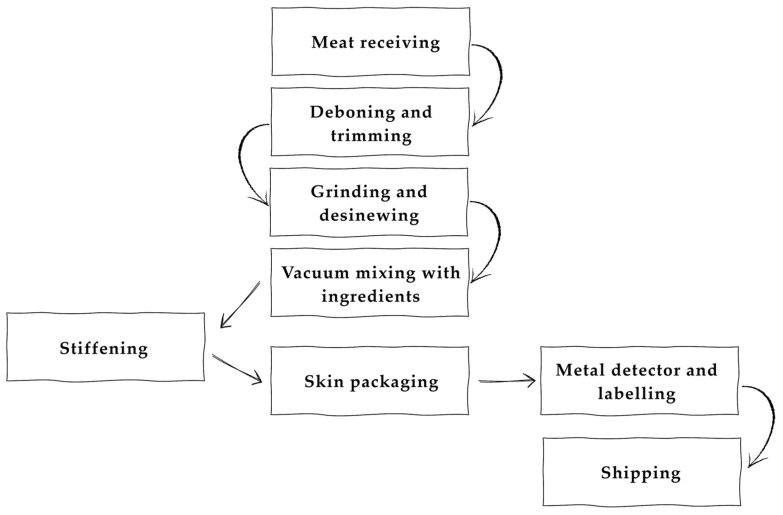
Flow diagram of the steak tartare processing and packaging workflow (adjusted from Eurocarni [14]).

The main steps in the flowchart (Figure 2) are trimming, grinding or slicing, vacuum mixing, stiffening, and VSP. To the best of our knowledge, no publication regarding the industrial production of steak tartare exists in the scientific literature, and the only information that is publicly available derives from specialised magazines [14]. However, the steak tartare products that are currently available in the EU market are highly variable in terms of recipes, physical and chemical characteristics, and packaging conditions.

In a typical manufacturing plant, trimming is usually done manually. In this first step, most of the superficial connective tissue must be removed to prepare meat cuts for grinding. To this purpose, a manual removal of the epimysium (also called “silver skin”) is often coupled with a mechanical membrane-skinning machine to obtain the so-called prêt à découper (PAD) cuts, also known as peeled cuts.

Desinewing is another important operation, aimed at removing most of the sinew or internal connective tissue. It generally improves the textural properties of minced meat, and the grinding diameter depends on the concentration of connective tissue in the meat. Usually, meat that is desinewed with the 0.19 cm head exhibits superior tenderness compared to the 0.25 and 0.32 cm heads [15]. This is especially true for steak tartare, for which the texture at bite should be homogeneous and should not present any sort of bulkier and crunchier particles. For this reason, the meat grinders that are normally used for the industrial manufacturing of steak tartare are equipped with an in-built desinewing component.

After the first grinding, a second low-impact grinding/mixing operation incorporates the mix of the different ingredients into the resulting meat paste. Usually, in this step, nitrite is added, and therefore it is crucial to have a vacuum mixer machine to permit the initiation of “the curing action” given by this additive. In fact, the reducing environment obtained in the vacuum chamber favours the reduction of nitrite to nitric oxide and subsequent binding to myoglobin to form nitrosylmyoglobin pigments, which are responsible for the “cured” red colour. Ascorbic acid and/or ascorbate are also added as a pro-technological additive, as they react with oxygen, forming dehydroascorbate and thus reducing the amount of nitrite that can be oxidised to nitrate.

Some mixing machines are coupled with a forming unit; otherwise, this is a separate step. After forming, stiffening is performed in cooling cabinets to increase product resistance to the pressure applied by the VSP machine. Stiffening is sometimes referred to as crust freezing, because the cooling forms a superficial frozen layer of 2–4 mm, which allows the product to keep its shape during VSP [16]. Process parameters such as temperature used, time of resting, speed of the conveyor belt, etc., are decided according to the type of machinery used (e.g., linear tunnels, spiral tunnels, blast freezing units, etc.), the dimension of the product, and the company’s guidelines.

The product is ready for skin packaging once the stiffening process is complete. VSP is the technique used for high-value products, as it improves product presentation, prevents the formation of air pockets [17], reduces the formation of exudate, retards lipid oxidation, and avoids off-smells at the time of opening [18]. In this operation, the steak tartare is deposited on a plastic or cardboard tray, which is lidded by a shrink-wrap film that adheres to the product and is sealed along the entire surface of the tray. Packaged steak tartare is then checked by a metal detector, labelled, packaged in carton, palletised, and stored at 0–2 °C until shipment.

By and large, the manufacturing process involves significant changes according to product formulation. In fact, the ingredients added can influence the mixing and packaging process, particularly when important additives need to be reduced or substituted.

## 4. Formulation of Steak Tartare

A distinctive feature of steak tartare is the low-fat content, usually below 5%. To obtain this result, lean beef cuts are used, such as shoulder and foreleg from forequarters, and thick flank and silverside for hindquarters. The choice of the meat cuts, together with other features such as breed, age, and maturation, is essential to obtain the desired colour, tenderness, juiciness, and overall acceptance. Nejedli et al. [19] investigated the different muscle fibre composition, colour, and flavour of steak tartare made from different muscles: m. triceps brachii, m. longissimus dorsi, m. psoas major, and m. semimembranaceus. The breed utilised was Croatian Simmenthal, and the animals were fattened bullocks, aged 15 months and weighing about 400 kg. The authors concluded that m. longissimus dorsi and m. psoas major were more fit for the preparation of steak tartare, given their lesser quantity of connective tissue, higher ratio of white fibres in respect to red fibres, and their redder colour in respect to the other two muscles [19].

In Italy, different cuts may be suitable for tartare and battuta, considering that the first has a tender texture, whereas the latter is crunchier and has a cubed appearance. In this respect, Abrati [20] suggested using cuts like tenderloin, sirloin, shoulder rump, topside, and bottom round for the preparation of steak tartare, as these cuts are tender although they have a duller colour and flavour (especially tenderloin). The same author suggested the use of firmer cuts, like the rump, tip of the rump, and shoulder spindle, for battuta. The cuts that display a brighter red are tip of the rump, heart of the rump, and topside. Moreover, the shoulder, rump, and internal part of the silverside make an important contribution to flavour, while the topside and heart of the rump are more delicate [20]. It is generally accepted in both industrial settings and butcher shops that the most suitable cuts for preparing steak tartare are the tenderloin, silverside, rump, and topside [21]. In the Italian steak tartare, the most used and reported breeds are Aberdeen Angus Sired, Chianina, Fassona Piemontese, Scottona Marchigiana, etc. [22,23,24,25].

Comparing the technical sheets available on the manufacturers’ websites, generally, the meat content of skin packed steak tartare ranges from 82 to 95%, pH values may range from 5.3 to 6, and a_w_ values from 0.97 to 0.99.

The product formulation includes a variety of additives and ingredients depending on the final product desired. Table 1, modified from Tirloni et al. [26], lists the most common formulations in the steak tartare marketed in Italy.

Water, salt, spices, and sugars (e.g., dextrose, sucrose) are added in almost all the products, while olive oil and other ingredients (e.g., PDO Parmesan cheese) are used in certain recipes [26]. Abrati [20] cited special ingredients (e.g., extra virgin olive oil, salt, pepper, rocket, and flakes of PDO Parmesan cheese) that are distinctively employed by certain producers.

The most common additives in the formulation of steak tartare are antioxidants (ascorbic acid/ascorbate), acidity regulators (mainly acetates), and preservatives (nitrite).

Heuvelink et al. [27] tested the effect of acetic acid, sodium lactate, a lactoperoxidase–thiocyanate–hydrogen peroxide system (a naturally occurring system in cow’s milk, which has been proven to be bacteriostatic to Gram-positive microorganisms and bactericidal to Gram-negative microorganisms), and storage conditions on the survival of VTEC O157. All the additives used did not prove any efficacy against the inoculum of *E. coli* O157, which was also resistant to cold temperatures (−20 °C) and acidic pH. These authors found that the inoculum growth was inhibited in the presence of a high aerobic plate count.

## 5. Microbial Ecology of Steak Tartare

In general, raw beef preparations contain a diverse microbial community that includes spoilage organisms and commensal microorganisms. Dominant species are usually lactic acid bacteria (LAB), *Pseudomonas* spp., *Brochothrix thermosphacta*, and *Enterobacteriaceae*, with significant changes depending on the manufacturing process and the packaging conditions, and a dominion of LAB in vacuum conditions and *Pseudomonas* spp. in aerobiosis [28].

To the best of our knowledge, the first investigation carried out on the microbiological quality of steak tartare was that of Beumer et al. [29], who analysed different preparations of Filet Américain (a mixture of minced meat, acid sauce, condiments, salt, etc., meant to be eaten raw), made of only beef or beef and pork. The microbial counts were generally lower in the samples containing pork. In detail, the aerobic plate count and the yeast count, as well as *Enterobacteriaceae* and group D streptococci, were at least 10 times higher on average in the samples not containing pork. In the case of *Lactobacillus* spp., *Staphylococcus aureus*, and *Clostridium perfringens*, the data were similar, though the counts of the latter two species were lower. *Salmonella* spp. was detected in 84% of the pork-containing samples and in 13% of the other samples. For *Yersinia enterocolitica*, these figures were 44% and 5%, respectively, and for *Campylobacter jejuni*, 18% and 6%, respectively.

Later, Roels et al. [30] investigated the causes of a large outbreak of *Salmonella* Typhimurium in Wisconsin in 1984 and found that the major factors involved were the incomplete sanitation of a meat grinder and the ingestion of raw ground beef.

Delhalle et al. [31], through quantitative real-time PCR (qPCR) assay used in combination with 16S rDNA metagenetics, identified up to 180 bacterial species and 90 genera in 58 samples of Belgian steak tartare with a shelf life of two days. The samples were classified according to the place of origin (butcher shops, restaurants, sandwich shops, and supermarkets) and the ingredients (only with acidity regulators or with a sauce). There were seven dominant bacterial species: *B. thermosphacta*, *Lactobacillus algidus* (now *Dellaglioa algida*), *Lactococcus piscium*, *Leuconostoc gelidum*, *Photobacterium kishitani*, *Pseudomonas* spp., and *Xanthomonas oryzae*. In the samples collected from restaurants and sandwich shops, there was a more diverse microbiota, comprising also *Streptococcus thermophilus* and *Clostridium haemolyticum*.

Regecová et al. [32] investigated the load of microbial contamination in tenderloins used for the manufacturing of steak tartare. They carried out experiments on four types of samples of tenderloin, each stored with a different storage condition before actually being used, and found an aerobic plate count that was 1 Log CFU/g higher in the products manufactured with tenderloins that had more days of storage, even if frozen (−18 °C, 21 days), compared to those made with fresh cuts. This result suggests the importance of using fresh meat for the manufacturing of steak tartare.

Tirloni et al. [33] found in samples of a 96% lean Scottona steak tartare that total viable counts (mesophilic and psychrotrophic) were around 4 Log CFU/g at the beginning of the study (t_0_) and reached values close to 7 Log CFU/g on the declared expiry date. LAB represented the main microbial group in the product, as expected for vacuum-packaged meat, with an evident increase during the 12 days-storage, from ~4 Log CFU/g to values above 8 Log CFU/g. *Pseudomonas* spp. were inhibited, and only a slow increase was observed, starting from an average of 3.10–4.50 Log CFU/g in 12-day storage at 4 °C. The same trend was found for *Enterobacteriaceae*, which showed a very limited increase, reaching values up to 4 Log CFU/g in 12 days of storage. The storage conditions applied inhibited *B. thermosphacta*, resulting in no growth throughout the experimental period. Yeasts did not grow in the conditions applied, while moulds and *Clostridium* spp. were always below the detection limit (2 and 1 Log CFU/g, respectively).

In another study, the same authors [34] analysed samples of a 95% lean veal steak tartare and found that the aerobic plate count at t_0_ ranged from 4.38 to 5.29 Log CFU/g. LAB were again the main microbial group, which increased from ~3 Log CFU/g to ~7 Log CFU/g during 12 days of storage. At t_0_, *Pseudomonas* spp. ranged between 3.08 and 4.08 Log CFU/g, whereas *Enterobacteriaceae*, *B. thermosphacta*, and yeasts were detected in very low counts during the experimental time, and moulds and *Clostridium* spp. were always below the detection limit until the end of the trial (2 and 1 Log CFU/g, respectively) [34].

Tirloni et al. [26], in another study on several brands of steak tartare marketed in Italy, confirmed that LAB were the main microbial group, with values exceeding aerobic plate count counts in some cases (brand J). According to the results, LAB seemed favoured when either the formulation contained cheese or it had a higher percentage of beef (e.g., brand J, 97% beef content, >6 Log CFU/g). Aerobic plate count was very heterogeneous among producers, with mean counts ranging from 4.16 (brand C) to 6.17 Log CFU/g (brand M). The brand C showed significantly lower values of aerobic plate count compared to other brands; it could be supposed that being the product with fewer ingredients, the contribution to the total count was lower. A moderate contamination by *Pseudomonas* spp. was observed, with mean counts ranging from 2.97 (brand K) to 4.11 Log CFU/g (brand J), while *Enterobacteriaceae* were present in most of the samples analysed, with mean values ranging from values near 2 Log CFU/g (brands D and K) to values close to 3 Log CFU/g (brands J and M). Yeast counts were detectable in most samples, with mean values ranging from 2.07 (brands F and G) to 3.38 Log CFU/g (brand L); finally, moulds were sporadically found (only products from six brands), with the highest mean value detected in brand C (3.03 Log CFU/g).

## 6. Pathogens and Outbreaks

Raw beef preparations and meat products containing raw beef, when consumed as RTE products, can potentially harbour a wide variety of pathogens, such as *Salmonella* spp., *L. monocytogenes*, and *Campylobacter* spp. [35]. In fact, slaughter and cutting hygiene, ingredient quality and shelf life, and cold storage are essential to protect steak tartare from contamination, growth and survival of pathogens, which can derive from raw materials or enter during processing. 

The Netherlands has an extensive history of outbreaks associated with the consumption of steak tartare. In this country, during September–November 2005, an important outbreak (169 reported cases) of *S. typhimurium* definitive phage type 104 (DT104) was linked to the consumption of contaminated beef, prepared as Filet Américain [36]. The strain involved in this outbreak was resistant to amoxicillin, chloramphenicol, sulphamethoxazole, tetracycline, and florfenicol. Moreover, the PFGE (Pulsed-field gel electrophoresis) and MLVA (Multiple-Locus Variable Number Tandem Repeat Analysis) types were indistinguishable from those observed in a DT104 outbreak (40 cases) in Denmark in August 2005 involving beef carpaccio [37]. The tracing started in September 2005 after a RASFF alert, which helped identify the contaminated beef shipment coming from an Italian supplier, also involved in the Denmark case [36].

A total of 23 cases of infection by an unusual phage type, *S. typhimurium* (Dutch) phage type 132 (ft132), were confirmed by laboratory diagnosis between 4 November and 30 December 2009 in the Netherlands. Once again, the exposure to this pathogen was due to the consumption of RTE meat products, in particular steak tartare [38].

Another important outbreak took place in Limburg, Belgium, and was caused by *E. coli* O157:H7, with 24 cases from 30 May 2012 to 15 July 2012, 17 of which were laboratory-confirmed; 5 cases developed Hemolytic Uremic Syndrome (HUS), and 15 were hospitalised. Also in this outbreak, one of the main causes was the consumption of steak tartare [39].

During September–October 2005, with 21 laboratory-confirmed cases and 11 probable cases, another important outbreak involving *E. coli* O157 was recorded in the Netherlands, caused by the consumption of steak tartare purchased either in a supermarket chain or at a butcher shop. In this outbreak, the beef supplier could not be traced [40]. Another important Danish outbreak with 20 laboratory-confirmed cases caused by *E. coli* in steak tartare took place from the end of December 2008 until the end of January 2009; the strain was characterised as serotype O157:H7, and *stx1*, *stx2*, *eae*, and *e-hly* positive [41].

Historically, the series of outbreaks caused by the consumption of steak tartare or similar products contaminated by *E. coli* O157:H7 began with the cases recorded in Oregon and Michigan from May to June 1982, when at least 47 people reported abdominal cramps, bloody diarrhoea, nausea, and fever in some cases [42]. In this outbreak, the vector of contamination was beef used for the manufacturing of hamburgers, in which the pathogen survived cooking in a fast-food chain. Although a similar sporadic case was recorded in 1975, the outbreak in 1982 was important to establish *E. coli* O157:H7 as a significant hazard in the consumption of beef [43]. In fact, back in those years, *E. coli* O157:H7 was an emerging infectious agent, and its first public health apparition was linked to the 1982 outbreak [43].

Nauta et al. [44] conducted an important risk assessment on VTEC in ground beef. Their exposure model predicted that approximately 0.3% of ground beef patties were contaminated with VTEC O157. Out of these contaminated patties, more than 60% were contaminated with 1 CFU only, and only 7% contained more than 10 CFUs. In the Dutch study, the probability of a single VTEC O157 cell resulting in illness was estimated to be approximately 0.5%, and around 1300 cases of gastroenteritis associated with VTEC O157 in steak tartare were predicted per year. Compared with the estimated total number of VTEC O157 cases (2000 cases) based on epidemiological data, it appears that a large fraction was associated with steak tartare consumption; however, there were large uncertainties regarding the estimates.

Between 2012 and 2018, 10 outbreaks of *S. typhimurium* and Newport were linked to the consumption of raw ground beef in the United States, resulting in 737 reported cases, 206 hospitalisations, and one death [45]. These outbreaks were associated with traditional dishes typically consumed raw, including Kibbeh (a Middle Eastern dish), Kitfo (an Ethiopian speciality), and “Cannibal sandwiches” (a traditional holiday dish popular in parts of the Upper Midwest) [45]. In 2019, raw ground beef was also implicated in outbreaks of Shiga toxin-producing *E. coli* (STEC) O103 across 10 U.S. states, leading to 209 reported cases and 29 hospitalisations [46]. Additionally, an outbreak of Enteroinvasive *E. coli* (EIEC) serotype O8:H19 in Thailand in 2023 was linked to the consumption of “larb-neua-dib,” a spicy raw minced beef salad. Genetic analysis revealed that the O8:H19 isolates closely resembled strains identified in the United States and the United Kingdom but were genetically distinct from the O96:H19 strains previously reported in Europe [47].

Another pathogen of great interest in steak tartare, given its high rate of hospitalisations and deaths, is *L. monocytogenes*. Tirloni et al. [26], using a predictive microbiology software, assessed the growth potential of *L. monocytogenes*, since the physical and chemical characteristics alone could not have been a sufficient hurdle for this microorganism. They found no growth in steak tartare samples, whichever brand was considered, at both temperatures (4 or 8 °C). A possible explanation could be the different additives used and the fact that the LAB counts were important.

In another study, Tirloni et al. [33] investigated the growth potential of *L. monocytogenes* throughout the shelf life (12 days) of steak tartare samples. According to the EURL (European Union Reference Laboratory) guidelines, the growth potential (δ) is calculated as the difference between the *L. monocytogenes* concentration found at the end and at the beginning of the shelf life; this method is important to classify the product as able or unable to support *L. monocytogenes* growth [48]. With the respective median values obtained from the three batches and having considered the worst ones (batches 1 and 2), a growth potential (δ = T12 − T0) of 0.38 Log CFU/g was obtained, indicating the absence of significant growth of the pathogen (δ < 0.5 Log CFU/g). Also, in this case, LAB were predominant in the steak tartare’s microbiota. Moreover, the authors stated that if proper hygiene is maintained during the production, contamination by *L. monocytogenes* load is normally lower than 1 Log CFU/g.

Stella et al. [34] investigated the growth of *L. monocytogenes* (strains coded 12MOB045LM, 12MOB085LM, and 12MOB089LM, which were isolated from meat and able to grow at low pH and low temperature) in veal tartare supplied by a medium-scale producer located in Northern Italy, with a total shelf life of 12 days. The challenge tests were conducted in triplicate at a temperature of 8 °C, simulating thermal abuse conditions. Also, in this case, with LAB being the predominant group in the product, even with an inoculation load of 1.40–1.88 Log CFU/g, a total maximum increase of +0.98 Log CFU/g was achieved after 10 days of storage (+0.33, +0.23, +0.22, +0.20, −0.03 for the time intervals t_0_, t_2_, t_5_, t_8_, t_10_, t_12_). Thus, also in this case, it seemed that LAB exerted an antagonistic effect against *L. monocytogenes* (also due to the pH decrease), and it was concluded that a starting concentration of at least 1.02 Log CFU/g (10 CFU/g) would have been necessary at t_0_ to overcome the threshold limit of 2 Log CFU/g. However, having witnessed a maximum increase of +0.56 Log CFU/g in batch 1 at t_5_, the authors stated that the product should be considered as supporting the growth of *L. monocytogenes*.

Hluchanova et al. [49] investigated the prevalence of *L. monocytogenes* in samples of vacuum-packed steak tartare from retailers in the Czech Republic and characterised the strains obtained. They found *L. monocytogenes* in 11 samples out of 20 from all tested producers, which indicates a prevalence of 55%; however, the enumeration of *L. monocytogenes* never exceeded the limit of 100 CFU/g at the end of shelf life. Molecular serotyping revealed different serotypes attributed to human listeriosis outbreaks (e.g., serotype 4b) and 1/2a, which has been the most frequent serotype associated with human cases of listeriosis in the Czech Republic between 2013 and 2020. As far as whole genome sequencing, sequence types ST29, ST37, ST451, and ST121 were found to be the most frequent; in particular, ST29, ST37, and ST451 were previously correlated with cattle abortion cases in Latvia during 2013–2018 as well as in a study on the genetic diversity of *L. monocytogenes* from dairy production in the United States. Moreover, ST121 is recognised as a sequence type present in persisting *L. monocytogenes* strains. This study provides the scientific evidence of the broad diversity of *L. monocytogenes* in vacuum-packed steak tartare [49].

As far as *Campylobacter* spp. is concerned, a poster presentation held in the “St. Louis American Center” [50], which was later published by the same authors [49], highlighted interesting insights and results for *Campylobacter jejuni* in steak tartare. *Campylobacter* species are microaerophilic and capnophilic (5% O_2_ and 10% CO_2_—some also require hydrogen) Gram-negative bacteria, mesophilic but able to survive at 4 °C, sensitive to low pH, and common etiological agents of gastroenteritis in many countries. *C. jejuni* is the major cause of campylobacteriosis in both humans and animals, which is characterised by diarrhoea and may result in severe diseases such as Guillain-Barré syndrome, an acute immunoinflammatory neuropathy. Kim et al. [51] modelled the kinetic behaviour of *C. jejuni* in beef tartare by fitting cell count into the Weibull model (primary model) for each temperature (4, 10, 15, 25, and 30 °C) and saw a lower level of decrease at lower temperatures. This was dependent on the expression of genes related to oxidative stress resistance (*sodB* and *katA*) and stress responses (*clpP*), which were higher in *C. jejuni* cells exposed to 4 °C than in cells exposed to 30 °C under both aerobic and microaerobic conditions. This confirmed the consumption of steak tartare as a risk factor for *C. jejuni* infection, as also stated by Doorduyn et al. [52]; moreover, it is important to note that this pathogen has an infectious dose as low as 5 Log of cells [53].

In addition to the western country preparation of steak tartare, other variants have been studied and investigated. An example is the Ethiopian Kitfo, made of raw minced beef mixed with powdered spices and butter. Tegegne and Ashenafi [54] evaluated the microbiological profile of 50 samples of raw Kitfo, collected from 10 different establishments (hotels, bars, and restaurants in Addis Ababa), and determined the growth potential of *Salmonella* spp. by inoculating a lab-made pasteurised Kitfo with *Salmonella* strains at an inoculum load of 2–3 Log CFU/g. All the raw Kitfo samples, from all food establishments, had aerobic mesophilic counts ranging from 2 × 10^7^ to 2 × l0^8^ CFU/g; coliforms, staphylococci, LAB, yeasts and moulds, and aerobic spores had counts of 4 Log, 6 Log, 5 Log, 4 Log, and 3 Log CFU/g, respectively. *Salmonella* spp. was isolated from 21 of the 50 raw Kifto samples collected from the various establishments, and the results of the inoculation test showed that the load of CFU/g obtained was 7 Log CFU/g within 12 h. This study demonstrates how poor hygiene practices and poor microbiological quality of the raw material can negatively influence the overall finished quality of steak tartare.

Tassew et al. [55] analysed Kitfo samples from 24 food establishments, environmental samples of cutting machines from the only slaughterhouse in Jimma Town, and carcass swab samples from butcher shops and from the same slaughterhouse. From the 165 samples collected, 10 different genera were isolated. The following number of isolates were found: *Proteus* spp. 89 (53.9%), *E. coli* 44 (26.6%) (37 of which were thermotolerant), *Providencia* spp. 23 (13.9%), *Citrobacter* spp. 15 (9%), *Pseudomonas* spp. 9 (5.5%), *Klebsiella* spp. 2 (1.2%), *Enterobacter* spp. 2 (1.2%), *Salmonella* spp. 2 (1.2%), *Shigella* group-A 1 (0.6%), and *St. aureus* 20 (12.1%). Of the two *Salmonella* isolates, the one found in a carcass was resistant to cephalexin. The single *Shigella* isolate was resistant to co-trimethoxazole, tetracycline, streptomycin, and trimethophrim. All *St. aureus* isolates were tested for antimicrobial resistance; the results showed that 90% were resistant to oxacillin, 85% to ampicillin, 65% to erythromycin, 60% to amoxicillin, 35% to streptomycin, and 20% to vancomycin. However, all the isolates were sensitive to cotrimoxazole, and 18 out of the 20 isolates were Methicillin-Resistant *St. aureus* (MRSA). In this study, the authors were not able to identify the origin of the microorganisms isolated from the samples.

Eshetea et al. [56] highlighted Kitfo as a potential reservoir of antibiotic-resistant bacteria. They evaluated the microbial load, diversity, and antibiotic resistance in Kifto samples taken from 26 randomly selected Ethiopian restaurants in the Washington metropolitan area. Aerobic plate count ranged from 4.30 to 7.08 Log CFU/g, with a mean of 5.88 Log CFU/g and, from a total of 96 isolates, the species found were *Pantoea* spp. (14%), *E. cloacae* (10%), *E. coli* (10%), *Hafnia alvei* (8%), and *Klebsiella oxytoca* (5%) for *Enterobacteriaceae* and *Pseudomonas luteola*, *P. florescens*, and *P. mendocina* for *Pseudomonadaceae*. Overall, 34 out of these 96 isolates were further characterised for their antibiotic resistance against cefazolin, cefoxitin, ampicillin, and nitrofurantoin. Of the 34 isolates, 25 (73%) were resistant to one or more antibiotics, with cefazolin (62%) and cefoxitin (50%) being the highest, followed by ampicillin (35%) and nitrofurantoin (18%). The percentages of resistance to each of the four antibiotics were as follows: *E. coli* (12.5%), *Enterobacter* spp. (100%), *Acinetobacter calcoaceticus* (100%), and *Serratia* (80%) for cefazolin; *E. coli* (25%), *Enterobacter* spp. (100%), and *A. calcoaceticus* (100%) for cefoxitin; *E. coli* (12.5%), *A. calcoaceticus* (100%), and *Klebsiella* spp. (80%) for ampicillin; *A. calcoaceticus* (100%) and *Serratia* spp. (60%) for nitrofurantoin. The *A. calcoaceticus* and *P. luteola* isolates were multi-drug resistant (MDR), because they were resistant to at least three classes of antibiotics.

One year later, Eshetea et al. [57] found a *Cronobacter sakazakii* strain in Kitfo samples, showing the presence of efflux pumps, such as AcrAB-TolC, AcrADTolC, and AcrEF-TolC, and antibiotic resistance genes, such as *FosA*, *marA*, *marB*, *MacB*, *MarR*, *gyrA*, and *gyrB*.

Table 2 presents the principal outbreaks occurring from 1982 to 2024 that are attributed to the consumption of raw or undercooked beef-based dishes.

## 7. Innovation in the Manufacturing Process of Steak Tartare

**Figure 3:**
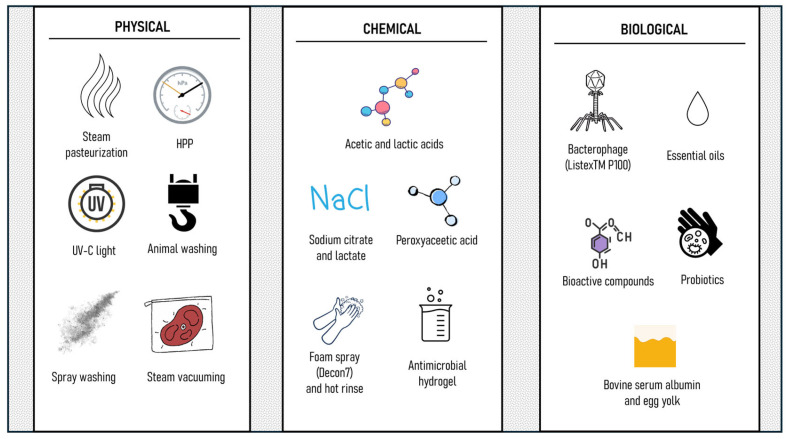
Overview of physical, chemical, and biological interventions in raw meat products processing.

In raw meat preparations, a wide set of technologies and approaches have been proposed to extend product shelf life and/or ensure food safety (Figure 3). These technologies include lactic acid rinsing, high-pressure processing (HPP), UV-C light, and natural antimicrobials [58,59,60]. From a microbiological point of view, Wheeler et al. [61] summarized the pre- and post-harvest interventions to be applied in the beef industry, as they identified three major gateways for pathogen contamination: (1) the load of pathogens contaminating the hides of animals; (2) the proficiency in hide removal that minimizes the transfer of contamination from the hide to the carcass; and (3) the effectiveness of antimicrobial interventions applied at various steps in the process. As for the last point, antimicrobial treatments can be physical, chemical, or biological.

Most studies on physical decontamination have focused on the steam pasteurisation of beef carcasses [62,63,64,65,66]. This method ensures the uniform coverage of carcass surfaces, even with complex geometries [66]. Following the 1996 *E. coli* O157:H7 outbreak in Lanarkshire, the Pennington Group recommended steam pasteurisation and improved HACCP protocols across the meat chain [67]. Tarkowski et al. [68] investigated the effects of decontamination by irradiation in steak tartare, determining the D values for different pathogens and concluding that a dose of 1 kGy was effective in inactivating the inocula of *Salmonella* spp., *Yersinia enterocolitica*, and *C. jejuni* without affecting the sensory characteristics of Filet Américain. Other physical decontamination treatments include live animal washing before slaughter, knife trimming, hot water washing, spray rinsing of the carcass with water or with solutions, steam vacuuming, etc. [69,70].

Non-thermal treatments, such as HPP, have been proposed and used in the industrial environment for steak tartare manufacturing. HPP can effectively reduce pathogens in steak tartare while maintaining product quality and flavour [71,72]. High-pressure processing is a non-thermal pasteurisation method widely used in the meat industry to ensure food safety and extend shelf life, with minimal impact on sensory and nutritional properties. It is particularly effective against pathogens such as *L. monocytogenes*, *Salmonella* spp., and *E. coli*, reducing the risk of foodborne outbreaks and product recalls [72]. However, in fresh, non-processed meats, HPP parameters (temperature, pressure, and time) must be carefully controlled, as they can alter the structure of sensitive proteins [72]. The first commercial HPP raw beef product was a steak tartare with spices, launched by Zwanenberg Food Group in the Netherlands in 2010. This product had a shelf life that was extended from a few days to 15 days, which significantly decreased *E. coli*’s risk [73]. Kameník et al. [74] determined the efficacy of HPP (400 or 600 MPa) compared with heat treatment in a water bath at 55 °C for inactivating STEC strains (O91, O146, O153, and O156) in samples of artificially contaminated steak tartare, also evaluating the effects on colour. The action of a pressure of 400 MPa resulted in a reduction in STEC of approximately 1.0–1.5 Log CFU/g, while 600 MPa caused a reduction of 6.0 Log CFU/g. The values of lightness L* and a* both increased compared to untreated steak tartare. The value of b* in the samples treated with a pressure of 400 MPa did not differ from the value recorded in the untreated samples, though the samples treated at 600 MPa showed a lower b* value than the untreated samples. After cooking in a water bath at 55 °C, the parameters of CIELab deviated in the same way as after HPP treatment, i.e., the value of L* and a* increased, and the value of b* decreased. The CIELab values in the samples treated with HPP and in a water bath did not differ from each other.

Among chemical treatments, several compounds have been reported in the literature, such as organic acids (acetic, ascorbic, citric, formic, fumaric, lactic, propionic acid, etc.), food-grade salts (sodium propionate, sodium lactate, potassium lactate, sodium citrate, sodium diacetate, etc.) [61,69,75]. Wang [76] investigated the presence of *S. enterica* in a meat processing plant and the efficacy of a sanitising agent used for an intense sanitisation (IS) procedure. In this study, the multicomponent sanitiser Decon7 (Decon7 Systems Inc., Coppell, TX, USA) was applied as a foam left in contact with surfaces for 8 h, followed by rinsing with 120–125 °C water and a final antimicrobial treatment with 200 ppm peroxyacetic acid.

Moreover, Oh et al. [77] developed several antimicrobial hydrogel formulations with edible polymers and antimicrobials (0.1% grape seed extract or 0.1% citrus extract) to improve the food safety of Yukhoe (Korean beef tartare); in this study, a reduction of 1 Log CFU/cm^2^ was observed on *L. monocytogenes* surface cell counts.

Finally, Botta et al. [78] investigated the decontamination potential of electrolysed water (EW) at 100 ppm of free chlorine for 90 s, used in a processing line for steak tartare to dip meat trimmings before grinding. Although the superficial aerobic plate counts decreased by 1 Log CFU/cm^2^ immediately after treatment, its efficacy was defied after grinding. This evidenced the poor suitability of this approach for overall improvement of the microbiological quality of steak tartare and for shelf-life extension, even though the chlorate content of the meat was under the detection limit (0.01 mg/kg), and its colour was not affected after grinding. However, a high microbial diversity was found between the two batches investigated, including *Streptococcus*, *Stenotrophomonas*, *Achromobacter*, *Lactococcus*, *Pseudomonas*, *Photobacterium*, *Luteibacter*, *Lactobacillus*, *Abiotrophia*, and *Neisseria*. One batch had more LAB of the genera *Lactococcus-Streptococcus* and Gram-negative genera *Luteibacter-Neisseria*, while the other had *Shigella* and *Burkholderia*.

In addition to physical and chemical treatments, different strategies based on the use of microorganisms as probiotics and prebiotics in feedstuffs have been evaluated [79,80].

Among biological interventions, Hluchanova et al. [49] investigated the effectiveness of ListexTM P100 against *L. monocytogenes*, artificially inoculated into steak tartare samples. P100 is a phage that is commonly used to combat *L. monocytogenes* and was originally isolated from the wastewater of a dairy plant. Twelve samples of steak tartare were inoculated with a sensitive isolate of *L. monocytogenes* LV1242, and another twelve samples with a resistant isolate of *L. monocytogenes* LV830 were used to reach a load of 3 Log CFU/g in the samples. In the first experiment, the concentration of P100 used was 8 Log PFU/g, which was not effective against sensitive isolate LV1242 and resistant isolate LV830 at all; in the second, it was 9 Log PFU/g, and a slight reduction in the count of sensitive isolate LV1242 was observed after 1 h of treatment, but it did not affect LV830 at all. The result is concordant with the suggestions of EFSA [81], which states that treatment with this product should only be considered as an additional tool following the application of Good Hygienic Practices (GHP) and Good Manufacturing Practices (GMP), since it does not guarantee the complete inactivation of *L. monocytogenes*.

Veldhuizen et al. [82] elucidated the inhibiting effect of both bovine serum albumin (BSA) and egg yolk on carvacrol, a major component of *Thymus vulgaris* and *Origanum vulgare* essential oils, used in vitro and for the treatment of Filet Américain samples containing 70% ground beef and 30% mayonnaise-based sauce. No antimicrobial effect was observed in the product, whereas the in vitro study demonstrated that carvacrol worked less effectively at lower temperatures and that BSA and egg yolk reduced their activity, even at a concentration of 2.5 mM. This is an important result, since it demonstrates the limitations of applicability of an important and known antimicrobial if used in the steak tartare formulation [82].

## 8. Nitrite in Steak Tartare: Significance and Possible Substitution

Nitrates and nitrites are nitrogen oxides that have received considerable attention for the preservation of animal products since the 19th century. Their usage is widespread among meat products for quality enhancement, microbial stability, and shelf-life extension. Currently, the use of nitrite in raw meat preparations and products is considered very important, if not essential, to control meat colour and the risk of *Clostridium botulinum* toxins formation.

Myers et al. [83] determined the effect of adding nitrite and HPP on the growth of *L. monocytogenes* in RTE sliced ham. A synergistic effect between HPP and nitrite concentration was observed; in fact, starting with a 3 Log CFU/g count after inoculation, the 200 ppm nitrite group reached a 7.57 Log CFU/g on day 21, without HPP; a 7.62 Log at day 91, with 400 MPa; and −0.69 Log at day 182, with 600 MPa. Moreover, HPP gave a significant reduction in the days following the treatment, whereas the only addition of nitrite did not inhibit *L. monocytogenes*. This finding is concordant with the fact that nitrite provides some inhibition on *L. monocytogenes* but is not listericidal.

Cropp et al. [84] evaluated the anti-*Listeria* potential of a nitrite-embedded film (NEF) used to pack nitrite-free bologna, evaluated after thermal processing. This film was a novel packaging solution (FreshCase^®^, Curwood Inc., Division of Bemis Company Inc., Neenah, WI, USA), consisting of a three-layer material (outer layer, a middle barrier layer, and a sealant layer) containing sodium nitrite crystals made with a nitrite-containing resin, and was the same as that used by Claus and Du [85]. The authors found that under their experimental conditions, even with an inoculation of 3.50 Log CFU/cm^2^, NEF was more effective at suppressing the growth of *L. monocytogenes* than nitrite in product formulation. In fact, at 50 days, the control group with conventional packaging and nitrite reached 7 Log CFU/cm^2^, while NEF samples had a 4 Log CFU/cm^2^ count. Moreover, the usage of NEF resulted in a lag time extension of at least 25 days.

Although early studies have shown that nitrite has some inhibitory effect on *L. monocytogenes*, the latest research has found that its effectiveness may vary depending on the strain [86]. As a matter of fact, the susceptibility of *L. monocytogenes* to nitrite is influenced by a combination of intrinsic and extrinsic factors. Genetic variability among strains plays a significant role, as differences in genomic profile can modulate resistance and tolerance mechanisms [87], even under mild acid and salt stress conditions encountered in raw meat products [88]. Consequently, the bacterium’s ability to tolerate nitrite exposure can change. In addition, factors like pH, salt concentration, and temperature can greatly affect how well nitrite works as an antimicrobial, either making it more effective or less effective against *L. monocytogenes* [89]. Other authors have investigated the effectiveness of nitrite on other foodborne pathogens, such as *S.* Thyphimurium and *St. aureus* [90,91], and spoilage microorganisms, such as *Lactobacillus* spp., *Pseudomonas* spp., and *Enterococcus* spp. [92,93,94]; moreover, the bactericidal mechanisms of nitrate and nitrite in cured meats have been comprehensively reviewed by Majou and Christieans [95]. Most importantly, nitrite has a key role in the inhibition of *Cl. botulinum*. According to Hustad et al. [96] and Bowen et al. [97], the incidence of toxicity declines sharply above the level of 50 μg of nitrite per g of product for type A and type B *Cl. botulinum* strains. Keto-Timonen et al. [98] demonstrated that nitrite included in the formulation of different vacuum-packaged meat products (Finnish wiener-type sausage, bologna-type sausage, and cooked ham) was able to limit the growth and inhibit toxigenesis of group II *Cl. botulinum* strains Eklund 2B, Eklund 17B, and Hatheway 706B. Group II *Cl. botulinum* members are psychrotrophic and thus can grow and produce toxins even at refrigeration temperatures. A significant growth of Group II *Cl. botulinum* was observed only in nitrite-free samples, which also had evidence of toxigenesis after 3 or 5 weeks of storage at 8 °C, while none of the products containing either 75 or 120 mg/kg nitrite became toxic during this storage period. This result suggests that the spores that survived the heat treatment were unable to germinate and produce toxin in the presence of nitrite.

In addition to its antimicrobial activity, nitrite expresses important technological functions in cured meat, e.g., by contributing to the colour in cured meats through the formation of nitrosyl-myoglobin and to the oxidative stability of lipids, where nitrite plays an antioxidant role through different mechanisms [99].

Nitrates and nitrites are regulated by Regulation (EC) No. 1333/2008 [100] on food additives. For a long period, the maximum level allowed in the EU for non-thermally treated meat products was 150 mg/kg for both nitrites and nitrates (expressed as NaNO_2_ and NaNO_3_, respectively), calculated as added during manufacturing. The maximum levels of nitrites (E 249 and E 250) and nitrates (E 251 and E 252) in foods were mainly based on the opinion of the European Food Safety Authority of 26 November 2003 [101]. According to the Authority, the added quantity of nitrites, rather than the residual amount, exerts a concentration-dependent antimicrobial effect in cured meat products, including the inhibition of the outgrowth of *Cl. botulinum* spores.

However, in 2017 and 2023, the safety of nitrites and nitrates as food additives was re-evaluated by EFSA [101,102]. In fact, two scientific opinions confirmed the relationship between dietary nitrite and gastric cancer, and the association of nitrite plus nitrate from processed meat with colorectal cancer. Moreover, several studies suggest that nitrite or nitrate additives are involved in different cancer risks via N-nitroso-compound formation (NOCs) [103]. Therefore, ‘meat and meat products’ are now considered the main food category contributing to the exposure of carcinogenic nitrosamines. Consequently, Regulation (EU) 2023/2108 was published. It amends Annex II to Regulation (EC) No 1333/2008 [100] by lowering the maximum limit of allowed nitrites and nitrates and changing how to express it; starting from 9 October 2025, it will be set to 80 mg/l or mg/kg for nitrites and 90 mg/L or mg/kg for nitrates, and will be expressed as NO_2_ ions and NO_3_ ions.

Consequently, the important regulatory changes concerning the use of nitrites and nitrates in meat products are likely to accelerate nitrite replacement in steak tartare formulations.

## 9. Current Challenges and Future Perspectives

The evolution of EU regulations on food additives, along with the growing consumer interest in clean label products that contain few ingredients and no additives, is prompting a switch towards nitrite-free meat products. Different nitrite-free meat products have been launched on the market, especially among cooked products such as cooked ham. At the beginning, most of the nitrite-free formulations were based on the addition of vegetable ingredients containing nitrates (vegetable-based nitrates) [104], such as celery juice or spinach powder. However, at least in Italy, since 2016, according to Ministerial Decree 26 May 2016 [105], these ingredients are considered food additives and cannot be used for products that are labelled as nitrite-free. Currently, other options are available for nitrite substitution in meat products to preserve colour and inhibit the growth of undesired microorganisms like *Cl. botulinum* while meeting consumer demand for clean labels. Different reviews have been published on nitrite substitution in meat products [106,107,108,109,110,111].

Among all the possible solutions reported in the scientific literature, most of the current industrial nitrite-free products are obtained by substituting nitrite with microbial cultures and/or plant extracts. In Italy, the first nitrite-free battuta, where nitrite is substituted by a combination of microbial cultures and plant extracts, has recently been launched on the market [112].

Different microbial cultures are available for potential applications as nitrite replacers in meat products, such as coagulase-negative staphylococci with NO-synthase activity [113].

Additional protective cultures have been investigated for their ability to replace nitrites and for their inherent antimicrobial activity. In particular, LAB such as *Latilactobacillus sakei*, *L. plantarum*, *L. curvatus*, *Lactococcus mesenteroides*, and *L. lactis* have been extensively studied for their effectiveness in controlling *L. monocytogenes* and *Pseudomonas* spp. in meat products, due to their GRAS status and ability to produce bacteriocins such as nisin and pediocin, while also contributing to the reduction of nitrite content [114,115,116,117,118]. Van der Veken et al. [119] conducted challenge tests on nitrate/nitrite-free fermented sausages using various starter cultures under different acidification conditions. While *C. botulinum* outgrowth was limited, *Mammaliicoccus sciuri* IMDO-S72 showed no anticlostridial effect, likely due to competitive inhibition. In contrast, *L. sakei* CTC 494 may have contributed to inhibition, possibly through bacteriocin (sakacin K) production.

A recent study raised the prospect of combining natural plant-derived extracts with denitrifying microbial cultures as an effective alternative to sodium nitrite [120]. This approach has shown promising results in enabling the complete replacement of nitrites in dry-cured meat products while maintaining colour stability and microbial safety, which are key parameters for ensuring product quality and consumer acceptance [120]. Moreover, several commercial mixtures of plant extracts and antioxidants have been proposed for the development of nitrite-free raw meat products. However, all these products need to be carefully checked for the possible presence of nitrite residues derived from raw materials.

Natural preservatives, such as grape seed extract, have been shown to reduce the use of nitrites in steak tartare, although they may influence the product’s colour and flavour [121]. Grape seed extract represents a promising alternative to conventional antioxidants, effectively decreasing oxidative rancidity and extending shelf life [121]. Its effectiveness mainly comes from its high content of proanthocyanidins, primarily monomeric catechin and epicatechin, gallic acid, and both polymeric and oligomeric forms, which exhibit potent free radical scavenging activity, surpassing that of traditional antioxidants such as vitamins C, E, and β-carotene [122]. Additionally, studies have reported that grape seed extract significantly reduces the natural bacterial community during storage [123].

Porto-fett et al. [124] utilised liquid buffered vinegar (LBV), dry buffered vinegar (DBV), or a blend of potassium lactate and sodium diacetate (KLac) at different concentrations in pork mortadella to investigate the effects against a cocktail of five strains of *L. monocytogenes*. The authors found that the ingredients used were effective in reducing *L. monocytogenes* count at 4 °C but not at 12 °C, with a load decrease of ca. 0.7 Log CFU/g after 120 days using KLac, ca. 0.3 or 0.5 Log CFU/g with 1.0 or 1.5% LBV, respectively, and ca. 0.6-Log CFU/g decrease with the inclusion of 1.0% DBV. Among the different types of vinegar, DBV had a higher cost but was more advantageous because it could be mixed with other ingredients, thus reducing formulation errors.

Roila et al. [125] assessed the antioxidant and anti-clostridial effects of two different polyphenolic extracts derived from olive mill vegetation water, in liquid form (LE) and encapsulated (EE), against *C. perfringens*, *C. botulinum*, and *C. difficile*. From an initial load of around 5 Log CFU/mL, both treatments were able to reduce the counts of each microorganism under the limit of quantification (LOQ), with LE being more efficient than EE and *C. perfringens* being the most sensitive species [125].

In another study, Strzelecki et al. [126] summarised the antimicrobial mechanisms of phytochemicals, such as isothiocyanates (ITCs), phenolics, terpenes, and terpenoids, against foodborne pathogens like *E. coli* O157:H7 in foods including beef. Dufour et al. [127] reported that ITCs can achieve antimicrobial effects at lower doses than synthetic agents due to potential synergistic interactions with other phytochemicals or antibiotics that reduce the establishment of antimicrobial resistance. Similarly, Nadarajah et al. [128] demonstrated that allyl isothiocyanate (AIT) effectively reduced *E. coli* O157:H7 in ground beef (1 to >3 Log CFU/g), with dose- and storage-dependent behaviour. Meira et al. [129] assessed the antimicrobial synergy of essential oil compounds (EOCs) and phenolic acids (PAs) against *E. coli* O157:H7 in vitro and in dry-fermented sausages. The combination of AIT and o-coumaric acid achieved ≥5 Log CFU/g reduction after 21 days.

Moreover, Tosi et al. [130] tested propolis extracts rich in phenolic compounds against *E. coli* ATCC 25922, observing inhibition at a mean concentration of 14.3 ± 6 mg/mL on cultures with 10^5^ CFU/mL. Notably, extracts from ginger (*Zingiber officinale* Rosc.), lemon (*Citrus limon*), pineapple (*Ananas sativus*), pomegranate (*Punica granatum*), pear (*Pyrus communis*), rose petal (*Rosa damascena*), and rosemary leaf (*Rosmarinus officinalis*) have demonstrated antimicrobial effectiveness against *Pseudomonas* spp., *L*. *monocytogenes*, *Enterobacteriaceae*, LAB, and naturally present bacteria [131,132,133]. These compounds have also been shown to significantly inhibit lipid and protein oxidation, with a performance comparable to, or even exceeding, that of synthetic additives [131,132].

Table 3 lists the natural preservatives proposed for nitrite replacement in meat products, as well as their active compounds and technological effects.

In the specific case of steak tartare and battuta, the development of nitrite-free formulation is particularly challenging and relies on the combination of several hurdles: ingredient quality, strict cold chain management, product uniformity, skin packaging, natural antimicrobials and antioxidants, and microbial cultures. Three main requirements need to be met: the control of meat colour along the shelf life (at least 10 days), the inhibition of *Cl. botulinum* toxins formation, and the control of *L. monocytogenes*. To achieve these goals, the experimental design for developing the novel formulations should consider any potential synergistic or antagonistic effects among the available technological solutions aimed at achieving each specific target. At the same time, to ensure the safety of future consumers of nitrite-free steak tartare, there is an urgent need to expand the knowledge on the microbial ecology of nitrite-free formulations containing microbial cultures, evaluating the microbial dynamics under different storage conditions and under thermal abuse.

In this respect, several advanced technologies have been implemented to ensure effective control and management of the cold chain, including combined chilled/frozen storage systems, superchilling, and active packaging. Moreover, the integration of wireless sensing devices with software-driven cold chain management platforms enables real-time monitoring and traceability of storage conditions throughout the entire distribution process [134]. Packaging also plays a pivotal role in the meat industry, contributing not only to the preservation of product quality and safety but also to extended shelf life. Thermoforming packaging and skin packaging are extensively used for steak tartare to shape packaging directly on the production line [135]. Compared to conventional systems, such as vacuum pouches and expanded polystyrene trays, thermoformed plastic films effectively limit the transmission of atmospheric gases, particularly oxygen, thereby improving the surface colour stability of fresh meat [135].

In Italy, a meat processing company [136] has achieved a 15-day shelf life in its 97% beef tartare by combining conventional additives (sodium nitrite, sodium acetate, sodium citrate, and sodium ascorbate) with natural ingredients (vegetable extracts, beetroot powder, and flavourings).

Finally, further studies should be conducted on steak tartare technology to highlight important chemical and physical features (i.e., hardness, firmness, drip loss, soluble protein content, myoglobin (Mb) and metmyoglobin (MetMb) content, etc.) and provide technical information for both manufacturers and consumers. On balance, consumers’ demand for safer, nutritious foods and clean label formulations is pushing innovative trends in food processing, which require new studies and interventions. Most probably, the answer to nitrite substitution will not be a single revolutionary molecule but a synergistic effect between more sustainable livestock production, targeted choice of the raw materials, innovative technologies during processing, alternative ingredients, and microbial starters.

Overall, the research on nitrite replacement in raw meat products could reshape the future of the meat market, opening new perspectives for premium-price products. In fact, different studies demonstrate that a large proportion of consumers are willing to consume clean label products [137] with interesting premium prices, depending on the information given on the label [138].

**Figure 4:**
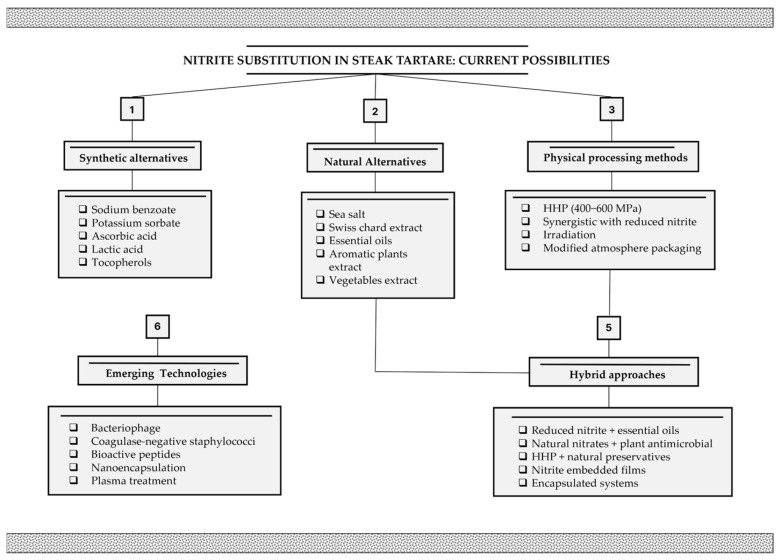
Comprehensive overview of nitrite substitution approaches in steak tartare: current alternatives and technological challenges.

Possible approaches for the replacement of nitrites in beef tartare, which have been described in this section, are summarised in Figure 4.

## 10. Conclusions

Steak tartare is consumed and appreciated worldwide, notably in Europe and Asia, and similar products are consumed in Africa and America. Only a few studies are available on raw meat products and preparations, whose chemical, physical, and microbiological characteristics seem far from standardised even in the same manufacturing plant, as indicated by the scientific literature. Even the technological solutions used for manufacturing industrial products show considerable variations, with some products that resemble the original steak tartare and others that appear crunchier, like the Italian battuta.

The market shows a clear trend towards nitrite-free formulations, which need to be safe without compromising product authenticity. Therefore, future research should focus on the development of nitrite-free formulations and the consequent dynamic changes in microbial ecology and product shelf life. Indeed, the development of novel formulations is challenging, because the presence of raw meat in an RTE product poses specific risks that need to be addressed by a combination of technologies and interventions. In this respect, future studies should provide scientific evidence on the safety of nitrite substitutes, as well as on the effects on foodborne pathogens such as *L. monocytogenes*, STEC, and *Cl. botulinum*. In these studies, collaboration among different disciplines will be essential to achieve the technological goals and ensure food safety.

On balance, the future evolution of steak tartare formulation and the probable substitution of nitrite with natural compounds and/or microbial cultures might act as a catalyst for the economic growth of the whole compartment, with promising perspectives for novel products at premium prices. For this reason, the information collected in this comprehensive review may serve as a basis for transforming the manufacturing process of raw meat products towards successful and sustainable targets.

## Figures and Tables

**Table 1:** Ingredients of different steak tartare brands based on manufacturers’ declaration (modified from Tirloni [26]).

Beef (%)	Other Ingredients	Antioxidants	Acidity Regulators	Preservatives
97%	Extra virgin olive oil, salt, spices (pepper, nutmeg)	-	-	Sodium nitrite
95%	Extra virgin olive oil 3%, salt, lemon juice, pepper	Sodium ascorbate	-	Sodium nitrite
93%	Water, salt, natural flavours, beetroot powder, paprika extract	-	-	-
92%	Extra virgin olive oil, water, salt, dextrose, pepper	Sodium ascorbate	Sodium acetate	Sodium nitrite
92%	Water, natural flavours, salt, sugars: dextrose	Ascorbic acid	Sodium acetates, sodium citrate	Sodium nitrite
91%	Salt, pepper, vegetable fibre, natural flavours, maltodextrin, sunflower oil, extra virgin olive oil	Ascorbic acid	Sodium acetates, sodium citrate	Sodium nitrite
91%	Extra virgin olive oil, water, flavourings, salt	Ascorbic acid, sodium ascorbate	-	-
91%	Extra virgin olive oil, water, flavourings, salt, pepper	Ascorbic acid, sodium ascorbate	-	-
88%	PDQ Parmesan cheese, extra virgin olive oil, salt, white pepper, dextrose	Sodium ascorbate	Sodium acetates	Sodium nitrite
85%	Water, salt, dextrose, fructose, sucrose, flavourings, black pepper, garlic	Ascorbic acid, sodium ascorbate	Sodium acetates	Sodium
85%	Water, salt, dextrose, fructose, sucrose, flavourings, white pepper	Ascorbic acid, sodium ascorbate	Sodium acetates	Sodium nitrite
85%	Water, salt, dextrose, fructose, sucrose, flavourings, black pepper, garlic	Ascorbic acid, sodium ascorbate	Sodium acetates	Sodium nitrite
82%	Water, cheese, salt, dextrose, natural flavours, sunflower oil, extra virgin olive oil	Ascorbic acid	Sodium acetates	Sodium nitrite

**Table 2:** Summary of reported outbreaks of foodborne pathogens linked to the consumption of raw or undercooked beef-based dishes (1982–2024).

Years	Country	Foodborne Pathogen	Food Product	Cases	References
1982	USA	*E. coli* O157:H7	Hamburger	≥47	[42]
2005	Netherlands	*S.* Typhimurium (DT104)	Filet Américain	169	[36]
2005	Denmark	*S.* Typhimurium (DT104)	Beef carpaccio	40	[37]
2009	Netherlands	*S.* Typhimurium (ft132)	Steak tartare	23	[38]
2012	Belgium	*E. coli* O157:H7	Steak tartare	24	[39]
2005	Netherlands	*E. coli* O157:H7	Steak tartare	21	[40]
2008–2009	Denmark	*E. coli* O157:H7	Steak tartare	20	[41]
2012–2018	USA	*S.* Typhimurium and Newport	Ground beef	737	[45]
2019	USA	*E. coli* O103	Ground beef	209	[46]
2024	Thailand	*E. coli* O8:H19	Minced raw beef salad	154	[47]

**Table 3:** Natural extracts as alternatives to nitrites in meat products: antimicrobial targets, active compounds, and technological effects.

Botanical Species	Main Active Compounds	Target Microorganisms	Technological Effects	References
Grape seed extract (*Vitis vinifera*)	Proanthocyanidins	Aerobic mesophilic count, fungi, yeasts	Reduces oxidative rancidity; extends shelf life; may affect colour and flavour	[120,121,122]
Ginger (*Zingiber officinale* Rosc)	Polyphenols, flavonoids	*E*. *coli* and *S*. Enteritidis	Antioxidant; antimicrobial; limits lipid and protein oxidation	[130]
Lemon (*Citrus limon*)	Ascorbic acid, flavonoids	*L. monocytogenes*	Antioxidant; antimicrobial; limits lipid and protein oxidation	[131]
Mustard oil (*Brassica juncea*)	Allyl isothiocyanate	*E. coli* O157:H7	Antioxidant; antimicrobial	[128]
Olive mill vegetation water (*Olea europaea*)	Phenolic compounds, tannin	*C. perfringens*, *C. botulinum*, *and C. difficile*	Antioxidant; antimicrobial	[124]
Pear (*Pyrus communis*)	Polyphenols, flavonoids	*Enterobacteriaceae* and *Pseudomonas* spp.	Antioxidant; antimicrobial; limits lipid and protein oxidation	[131]
Pineapple (*Ananas sativus*)	Polyphenols, organic acids, bromelain	*Pseudomonas* spp.	Antioxidant; antimicrobial; limits lipid and protein oxidation	[131]
Pomegranate (*Punica granatum*)	Ellagitannins, punicalagin, anthocyanins	*Pseudomonas* spp., LAB, and *L. monocytogenes*	Antioxidant; antimicrobial; limits lipid and protein oxidation	[131]
Propolis extracts	Phenolic acids, flavonoids, terpenoids	*E. coli* ATCC 25922	Antioxidant; antimicrobial	[129]
Rose petal (*Rosa damascena*)	Flavonoids, phenolic compounds, saponins, tannins, terpenoids	Aerobic mesophilic count, LAB	Antioxidant; antimicrobial; limits lipid and protein oxidation	[132]
Rosemary leaf (*Rosmarinus officinalis*)	Polyphenols, flavonoids, terpenoids	Aerobic mesophilic count, LAB	Antioxidant; antimicrobial; limits lipid and protein oxidation	[132]
Vinegar (Liquid buffered and dry buffered vinegar)	Flavonoids, phenolic acids	*L. monocytogenes*	Antioxidant; antimicrobial	[123]

## Data Availability

The original contributions presented in the study are included in the article. Further inquiries can be directed to the corresponding author.
